# *N*^6^-methyladenosine regulates RNA abundance of SARS-CoV-2

**DOI:** 10.1038/s41421-020-00241-2

**Published:** 2021-01-28

**Authors:** Ting Zhang, Ying Yang, Zichun Xiang, Chun-Chun Gao, Wenjing Wang, Conghui Wang, Xia Xiao, Xing Wang, Wei-Nan Qiu, Wen-Jie Li, Lili Ren, Mingkun Li, Yong-Liang Zhao, Yu-Sheng Chen, Jianwei Wang, Yun-Gui Yang

**Affiliations:** 1 CAS Key Laboratory of Genomic and Precision Medicine, Collaborative Innovation Center of Genetics and Development, College of Future Technology, Beijing Institute of Genomics, Chinese Academy of Sciences, Beijing, 100101 China; 2 China National Center for Bioinformation, Beijing, 100101 China; 3 University of Chinese Academy of Sciences, Beijing, 100049 China; 4 Sino-Danish College, University of Chinese Academy of Sciences, Beijing, 101408 China; 5 Institute of Stem Cell and Regeneration, Chinese Academy of Sciences, Beijing, 100101 China; 6 National Health Commission of the People’s Republic of China Key Laboratory of Systems Biology of Pathogens and Christophe Mérieux Laboratory, Institute of Pathogen Biology, Chinese Academy of Medical Sciences & Peking Union Medical College, Beijing, 100730 China; 7 Key Laboratory of Respiratory Disease Pathogenomics, Chinese Academy of Medical Sciences and Peking Union Medical College, Beijing, 100730 China

**Keywords:** RNA modification, Methylation

Dear Editor,

The worldwide pandemic of COVID-19 is caused by a novel β-coronavirus SARS-CoV-2, an enveloped RNA virus with a positive-sense, single-stranded RNA genome of ~30 kb^1,2^, which raises a key unresolved issue about its transcriptomic and epitranscriptomic architectures. The complete viral genomic RNA sequence contains six major open-reading frames (ORFs), including two large polyproteins ORF1a and ORF1b that can form nonstructural proteins through proteolytically cleaving upon cell entry. Other subgenomic mRNAs (sgRNAs) encoding structural proteins including spike protein (S), envelope protein (E), membrane protein (M), nucleocapsid protein (N), and accessory proteins (3a, 6, 7a, 7b, and 8), generated through a mechanism termed discontinuous extension of minus strands.

All the viral transcripts contain a 5′ cap, a common 5′ leader sequence of around 70 nt, a common 3′ untranslated region (3′UTR), and a 3′ poly(A) tail^3^. Moreover, the formation of sgRNAs is based on the discontinuous transcription leading to the leader–body fusion controlled by the RNA-dependent RNA polymerase and transcription-regulatory sequences (TRSs). TRSs, located at the 3′ end of the leader sequence (TRS-L) and preceding each viral gene (TRS-B), contain a conserved 6–7 nt core sequence and variable 5′ and 3′ flanking sequences^4^.

RNA chemical modifications are involved in physiology and pathology processes through regulating RNA metabolism. *N*^6^-methyladenosine (m^6^A) as the most abundant methylation type in mRNA has been shown to regulate the viral life cycles and the cellular response to viral infection^5,6^. Recently, dozens of RNA modification sites have been identified through nanopore direct RNA sequencing^7,8^, while the intrinsic nature and the detailed functions of the RNA modifications remain obscure. Here, we conducted m^6^A MeRIP-seq (methylated RNA immunoprecipitation sequencing) using RNAs from SARS-CoV-2-infected Vero cells, and identified 13 m^6^A-modified peaks on viral transcripts (11 peaks with the conserved eukaryotic motif RRACH (R = A/G; H = A/C/U)). We found that m^6^A might regulate abundance of SARS-CoV-2 through a mechanism of 3′UTR with or without RRACH.

We first performed strand-specific MeRIP- and RNA-seq for both positive-sense (SARS-CoV-2 RNA, +) and negative-sense RNA (SARS-CoV-2 RNA, −) to profile the m^6^A landscape along SARS-CoV-2 transcriptome. The results showed that more than 99.4% reads aligned to SARS-CoV-2 derived from positive-sense RNA and that only less than 0.6% from negative-sense RNA in both immunoprecipitated (IP) and input samples (Supplementary Fig. S1a). As the reads were predominantly aligned to the positive-sense RNA, and the number of reads for negative-sense RNA was not sufficient for identifying m^6^A peak, we only chose the reads aligned to the viral positive-sense RNA for subsequent analysis. To sensitively identify the m^6^A modifications in the viral RNA, the DAMS (Differential expressed window-based Analysis for MeRIP-Seq) algorithm was designed based on the analysis model reported in previous study^9^ (Supplementary Fig. S1b). To validate the sensitivity and specificity of DAMS in m^6^A peak calling, we first performed DAMS on the data derived from the host Vero cells and obtained 5822 m^6^A peaks belonging to *Chlorocebus sabaeus*. The motif from DAMS m^6^A peaks was conserved with the m^6^A feature (GACH) (Supplementary Fig. S1c). We also performed MACS2 (a common algorithm for peak calling) to identify the m^6^A peaks in host Vero cells and found that the methylome from DAMS was well coincident with that obtained from MACS2 (Supplementary Fig. S1d, e), suggesting that DAMS is a sensitive and specific algorithm in identifying m^6^A peaks.

We next performed DAMS on the viral transcriptome and finally identified 13 m^6^A peaks along the viral positive-sense RNA (Fig. 1a, b; Supplementary Table S1), which were all validated by MeRIP-qPCR (Supplementary Fig. S1f). Intriguingly, 9 of the m^6^A peaks (69.2%) were located in the CDS segments of ORF1ab (Fig. 1b), a much longer coding region than other parts of the genome sequence (Supplementary Fig. S1g). After normalization by the length, the segment of 3′UTR presented much higher m^6^A enrichment than ORF1ab (Fig. 1c), suggesting a potential regulatory role of m^6^A in post-transcriptional regulation of SARS-CoV-2. Moreover, we found 11 of 13 m^6^A peaks containing RRACH (Fig. 1d). To validate this result, we simulated the m^6^A pools by randomly shuffling peak locations for 1000 times and found that over 90% simulated pools (926 in 1000) contained less than 11 peaks with RRACH (Fig. 1e).

**Fig. 1:**
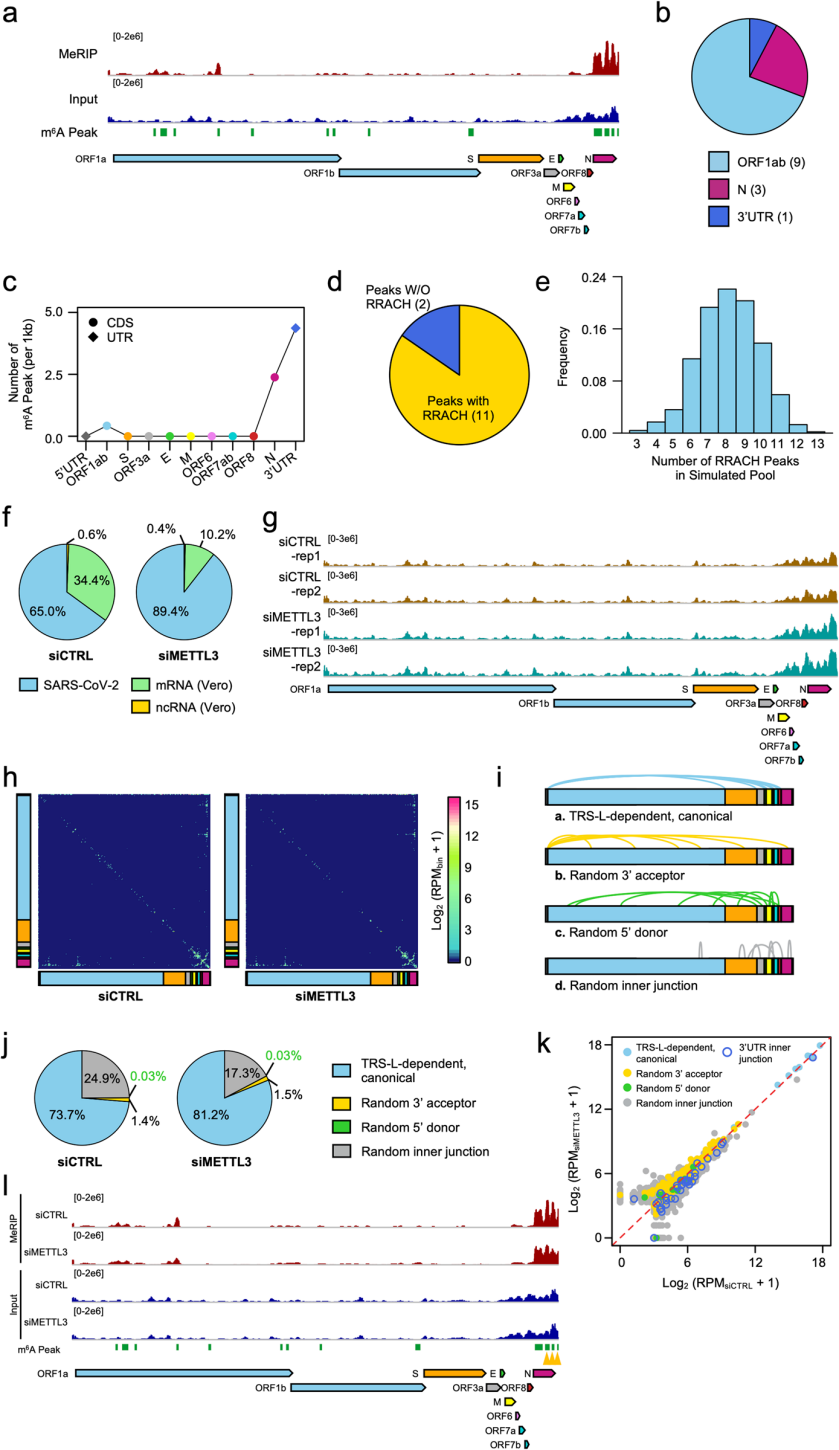
m^6^A methylome and diverse 3′UTR in SARS-CoV-2. **a** Integrative Genomics Viewer (IGV) tracks displaying read distributions from m^6^A MeRIP-seq (upper panel) and RNA-seq (lower panel) along SARS-CoV-2 positive-sense RNA. The green rectangles at the bottom depict the positions of identified m^6^A peaks. **b** Pie chart showing the fraction of m^6^A peaks in different segments along SARS-CoV-2 RNA genome. **c** Line chart revealing m^6^A peak numbers per 1 kb for each segment along SARS-CoV-2 RNA genome by normalizing with its length, respectively. The diamonds represent UTRs and dots represent CDS regions along complete SARS-CoV-2 genomic RNA. **d** Pie chart depicting the proportion of m^6^A peaks containing RRACH (yellow) or not (blue). **e** Histogram showing the frequency of RRACH peak number in 1000 simulated peak pools of SARS-CoV-2 genome. **f** Pie chart showing proportion of reads annotated as *Chlorocebus sabaeus* mRNA (green), ncRNA (yellow), and SARS-CoV-2 RNA (blue). **g** IGV tracks showing read distribution from RNA-seq along SARS-CoV-2 RNA in both control (upper panel) and METTL3-depleted Vero cells (lower panel). The read counts were normalized by ERCC for each batch, respectively. **h** Heatmap showing the frequency of junction-spanning reads along SARS-CoV-2 RNA genome from control (left) and METTL3-depleted Vero cells (right). The counts were aggregated into 100-nt bins for both axes. **i** Models present four different types of junction-spanning reads. (a) TRS-L-dependent, canonical fusion mediated by TRS-L and TRS-B; (b) Random 3′ acceptor represents noncanonical fusion mediated by TRS-L but not TRS-B; (c) Random 5′ donor represents long-distance fusion mediated by TRS-B but not TRS-L; (d) Random inner junction identified as TRS-independent spanning. **j** Pie chart showing the proportions of four different types of junction-spanning reads. **k** Scatterplot showing the relative expression levels of four different junction-spanning transcripts in control and METTL3-depleted Vero cells which were presented in different colors, respectively. The blue circle represents the spanning-junctions located in 3′UTR. **l** IGV tracks displaying read distribution from m^6^A MeRIP-seq along SARS-CoV-2 RNA genome in both control and METTL3 knockdown samples. The green rectangles depict the positions of m^6^A peaks identified in control samples and yellow triangles at the bottom represent peaks with significant decreased methylation level in METTL3 knockdown samples.

To investigate the regulatory role of m^6^A in the viral lifecycle, we first knocked down the METTL3, a key m^6^A methyltransferase subunit, in Vero cells infected with SARS-CoV-2 (Supplementary Fig. S2a), and performed whole transcriptome sequencing. Intriguingly, we found that the proportion of SARS-CoV-2 RNA in library was increased in METTL3-depleted Vero cells (Fig. 1f) and the increased abundance of viral RNA was validated by qPCR (Supplementary Fig. S2b), suggesting a suppressive effect of m^6^A on viral abundance. To quantify the absolute viral RNA in normal and METTL3 knockdown cells, we added ERCC (External RNA Controls Consortium) as an internal artificial reference for normalization during library construction (Supplementary Fig. S2c). The normalized results still showed an obvious increase in the viral RNA amounts in Vero cells upon METTL3 depletion (Fig. 1g).

As m^6^A has been reported to regulate RNA stability^10^, we next quantified the expression levels of all sgRNAs to further investigate the functional role of m^6^A in viral abundance. However, since the library only captured fragmented RNA and different viral sgRNAs with shared common regions, we can only count the spanning-junction reads harboring both leader sequence and CDS sequence for sgRNA quantification except ORF1ab. We first compared the counts of spanning-junction reads between two biological replicates and found that they are well conserved in both normal and METTL3-depleted Vero cells (Supplementary Fig. S2d). Besides, in both samples, the predominant proportion was spanning-junction sgRNA reads (with leader sequence and CDS) rather than other spanning-junction reads (non-defined). Nevertheless, we did not observe any significant changes in proportions of different categories of sgRNAs between normal and METTL3-depleted Vero cells (Supplementary Fig. S2d). Collectively, these results suggest a global increase in viral RNAs instead of increase in some specific sgRNAs.

We further investigated the 5′ and 3′ selections for each spanning-junction read. As expected, TRS-L and TRS-B were significantly enriched in 5′ and 3′ selections, respectively (Supplementary Fig. S3a, b). Besides, we also found a large amount of spanning-junction reads related to 3′UTR (Supplementary Fig. S3a, b). Based on our previous findings that m^6^A tends to be enriched in 3′UTR, we speculate that m^6^A may be involved in the regulation of 3′UTR spanning-junction. We then profiled the global pattern of spanning junctions and found a decreased signal around 3′ termini upon METTL3 depletion (Fig. 1h). Referring to a recent study by Kim et al.^7^, we defined the spanning-junctions into four groups (Fig. 1i; TRS-L-dependent, canonical; Random 3′ acceptor; Random 5′ donor; Random inner junction) and found that the proportion of random inner junctions was decreased by almost 8% (Fig. 1j). Through comparing the relative expression level of all spanning-junctions between control and METTL3-depleted Vero cells, we found that most 3′UTR inner junctions were decreased upon METTL3 depletion (Fig. 1k). Then, we performed MeRIP-seq in METTL3-depleted Vero cells, and found that the methylation levels of 3 m^6^A peaks near the 3′ termini (two located in sgRNA N, and one in 3′UTR) were significantly decreased upon METTL3 knockdown, while others kept a similar level of enrichment after depleting METTL3 (Fig. 1l; Supplementary Fig. S3c–e). We further mapped the RRACH motif in m^6^A peaks along SARS-CoV-2 genomic 3′UTR and found that most inner junctions locate in the vicinity of RRACH suggesting the possibility of RRACH “cut-off” in 3′UTR (shorter 3′UTR) (Supplementary Fig. S3f). Most regular 3′UTRs of viral RNA contain m^6^A-modified RRACH, which might promote the degradation of viral transcripts.

In summary, our work analyzed the m^6^A methylome of SARS-CoV-2 and suggests a potential regulatory role of m^6^A in SARS-CoV-2 RNA abundance, through shorter 3′UTR formation to evade the degradation of viral RNA. Erasing m^6^A through knocking down the host m^6^A methyltransferase METTL3 might decrease the diversity of 3′UTR as less spanning-junctions were identified. Thus, we propose that there might be two types of viral 3′UTR, with (shorter 3′UTR) or without random inner junction (regular 3′UTR). In normal cells, viral sgRNAs with regular 3′UTR can be methylated by the host METTL3, which likely stimulates the cellular degradation program to clear away the viral RNA. On the other hand, to resist the m^6^A-dependent degradation, SARS-CoV-2 might acquire diverse 3′UTR by depleting m^6^A-modified RRACH motifs (Supplementary Fig. S4). However, these perspectives need further investigations by using in vitro cell system and further in vivo animal models with an intact interferon system.

## Supplementary information

Supplementary Information

Supplementary Table S1

Supplementary Table S2

## Data Availability

The sequencing data have been deposited in the Genome Sequence Archive of National Genomics Data Center under the accession number CRA003003 linked to the project PRJCA003106 (http://bigd.big.ac.cn/gsa/s/gfL3V1dc).

## Supplementary information

**Supplementary Information** accompanies the paper at (10.1038/s41421-020-00241-2).
