# Supplementary material for: N6-methyladenosine regulates RNA abundance of SARS-CoV-2
Source: Cell Discov. 2021 Jan 28;7:7. doi: 10.1038/s41421-020-00241-2 (PMC7841760; doi:10.1038/s41421-020-00241-2)
Supplement: Supplementary file 1 — Supplementary Information [file 41421_2020_241_MOESM1_ESM.pdf]

Supplementary information, Figures

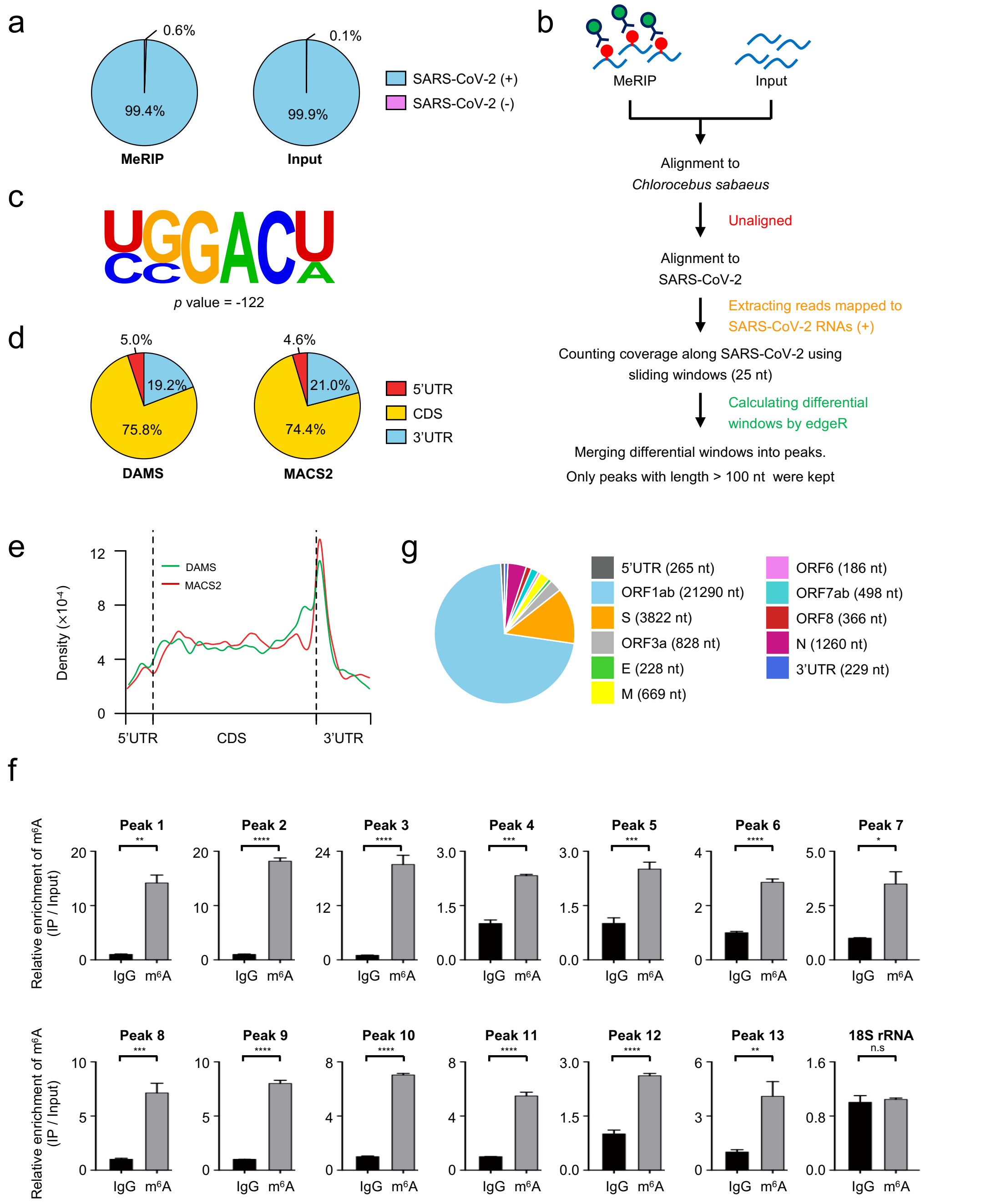

Supplementary Fig. S1. Identification of viral m^{6}A peaks using DAMS. a Proportion of reads distribution in positive-sense and negative-sense SARS-CoV-2 RNAs. **b** Schematics of peak calling and definition using DAMS for SARS-CoV-2. **c** Sequence motif of DMAS-m^{6}A-peaks in *Chlorocebus sabaeus*. **d** Pie chart showing the fraction of the annotation in three transcript segments of *Chlorocebus sabaeus* for DAMS-m^{6}A-peaks (left) and MACS2-m^{6}A-peaks (right). **e** Metagene profiles of m^{6}A

peak distribution along normalized *Chlorocebus sabaeus* transcripts composed of three rescaled non-overlapping segments (5'UTR, CDS, and 3'UTR) for DAMS-m^6A-peaks (green) and MACS2-m^6A-peaks (red). **f** m^6A MeRIP-qPCR assays showing the existence of m^6A in the thirteen peaks identified by DAMS. 18S rRNA serves as the negative control. Error bars, mean ± S.E.M. p values were determined using two-sided Student's t-test, *$p < 0.05$, **$p < 0.01$, ***$p < 0.001$, ****$p < 0.0001$. **g** Pie chart showing the proportions of segment lengths along SARS-CoV-2 genome.

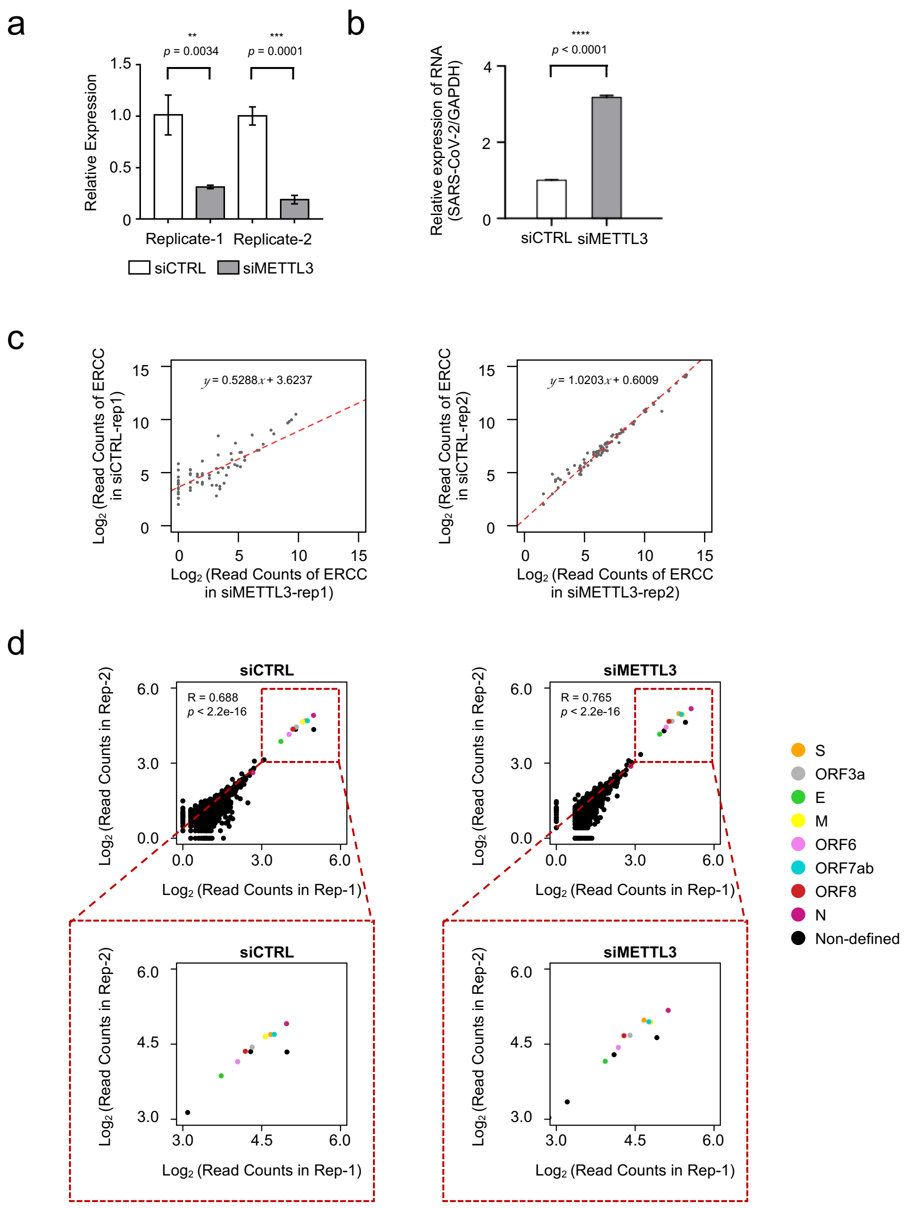

Supplementary Fig. S2. Knockdown efficiency and ERCC regression for sample normalization. a Expression of *METTL3* mRNA in control and siMETTL3 samples by RT-qPCR. Data are mean as ± S.E.M. *p* values were determined by a two-sided unpaired Student's *t*-test, ***p* < 0.05, *****p* < 0.0001. **b** Expression of SARS-CoV-2 in control and siMETTL3 samples by RT-qPCR. Error bars, mean ± S.E.M. *p* values were determined using two-sided Student's t-test, *****p* < 0.0001. **c** Scatterplot displaying the linear regression of ERCC read count between control and siMETTL3 samples. **d** Scatterplot depicting the different junction-spanning reads between two replicates of both control (left) and siMETTL3 (right) samples. The colored dots represent the TRS-

L dependent, canonical junction-spanning type (See Fig 1i), which were considered as the expression levels of different sgRNAs.

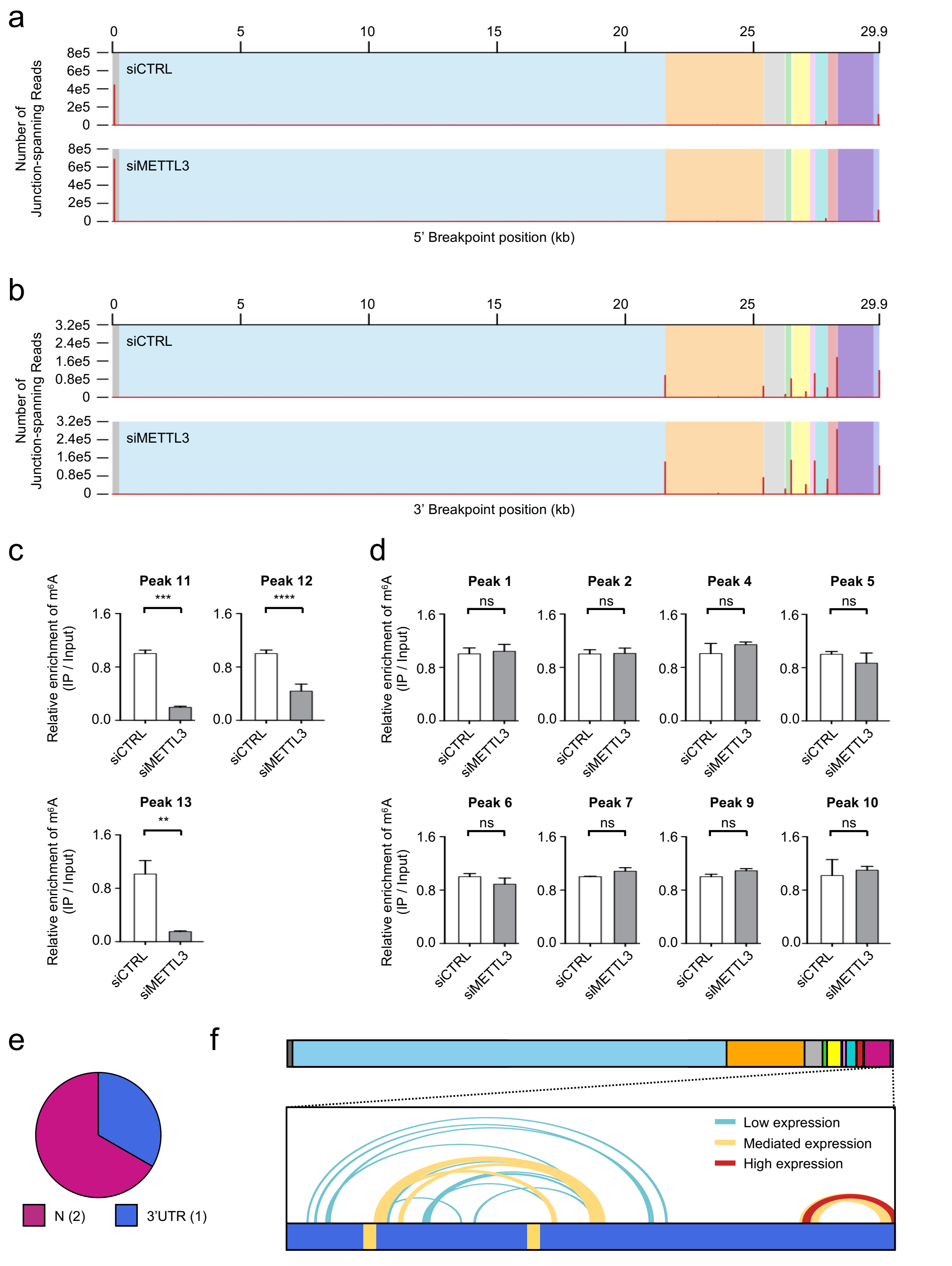

Supplementary Fig. S3. The inner junction-spanning in 3'UTR might lead to depletion of m^{6}As. a Junction-spanning reads coverage of 5' breakpoint position along positive-sense SARS-CoV-2 RNA. **b** Junction-spanning reads coverage of 3' breakpoint position along positive-sense SARS-CoV-2 RNA. **c,d** m^{6}A MeRIP-qPCR assays showing the enrichment changes of METTL3-dependent (**c**) and -independent (**d**) m^{6}A peaks identified in SARS-CoV-2 RNA upon METT3 knockdown. Error bars,

mean ± S.E.M. p values were determined using two-sided Student's t-test, $**p < 0.01$, $***p < 0.001$, $****p < 0.0001$. **e** Pie chart showing the annotated position of down-regulated m^6A peaks upon METTL3 depletion. **f** IGV plot showing the 5' and 3' position sites along 3'UTR of SARS-Cov-2 RNA for identified random 3'UTR inner junction. The blue rectangle at bottom represents the 3'UTR segment and two inner yellow rectangles represent two RRACH sites in the m^6A peak of 3'UTR. The different colors of circles represent the expression level of random 3'UTR inner junction (Blue: low expression junction with $log_2(RPM+1)$ less than 5; Yellow: mediate expression junction with $log_2(RPM+1)$ from 5 to 10; Red: high expression junction with $log_2(RPM+1)$ more than 10).

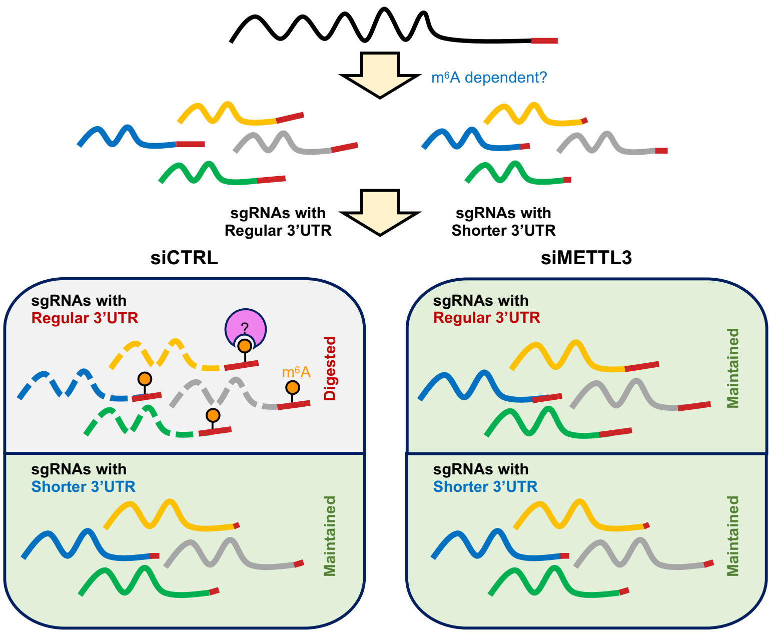

Supplementary Fig. S4. Schematic model of the m^{6}A regulates RNA abundance of SARS-CoV-2. Schematic model showing that the m^{6}A-dependent degradation invovles in the regulation of SARS-CoV-2 abundance via the m^{6}A in viral regular 3'UTR while the SARS-CoV-2 acquaired diverse shorter 3'UTR to evade it.

Supplementary information, Materials and Methods

Cell culture, siRNA transfections, and virus infection. Vero cells (ATCC, CCL-81) were cultured in DMEM (ThermoFisher) supplemented with 10% fetal bovine serum (HyClone, Logan, UT) and penicillin-streptomycin (ThermoFisher) at 37℃, 5% CO_2.

The double-stranded RNA oligonucleotides (short interfering RNA, siRNA) used for RNA interference were obtained from GenePharma. Lipofectamine RNAiMax (ThermoFisher) was used for transfection of siRNA duplexes according to the manufacturer's instructions. 48 hours post-transfection, cells were infected with SARS-CoV-2[1], at a multiplicity of infection (MOI) of 0.1, and were collected for RNA isolation 24 hours later. All experiments with the SARS-CoV-2 virus were performed in the BSL-3 laboratory. The siRNAs used in this study were listed in **Supplementary Table S2**.

RNA purification and rRNA depletion. Cultured cells were washed once with PBS and then subjected to RNA purification with TRIzol (ThermoFisher). Purified total RNAs were first treated with Turbo DNase (ThermoFisher) and then depleted the rRNAs with the Ribo-off rRNA Depletion Kit (Human/Mouse/Rat) (Vazyme, N406-02) following the manufacturer's instructions.

Quantitative polymerase chain reaction (qPCR). Total RNAs were used to generate cDNA by RevertAid™ First Strand cDNA Synthesis Kit (ThermoFisher, k1621) according to the manufacturer's instructions. qPCR was performed with ChamQ Universal SYBR qPCR Master Mix (Vazyme, Q711-02) following the manufacturer's instructions, and quantified on a Real-Time PCR System (Agilent). GAPDH was used as internal control. All primers used were listed in **Supplementary Table S2**.

RNA-seq. For RNA-Seq, 1 μg total RNA of each sample was mixed with 2 μl ERCC RNA Spike-in Mix 1 (1:100; ThermoFisher, 4456740) and then subjected to the rRNA depletion with the Ribo-off rRNA Depletion Kit (Human/Mouse/Rat) (Vazyme, N406-02). Libraries were constructed using the KAPA RNA HyperPrep Kit (KAPA

Biosystems, KK8541) following the manufacturer's instructions.

m^{6}A MeRIP-seq & m^{6}A MeRIP-qPCR. 100 ng of the purified ribominus RNAs were fragmented into a size of around 100 nt using RNA fragmentation reagents (ThermoFisher, AM8740), and then were mixed with 5 μg of anti-m^{6}A antibody (Synaptic Systems, 202003) and 20 μl Dynabeads Protein A (Invitrogen, 1001D) in 500 μl IPP buffer (150 mM NaCl, 10 mM Tris-HCl, pH 7.4, 0.1% NP-40) supplemented with 10 U RNase Inhibitor (Beyotime) and incubated at 4 °C for 4 h with rotation. After extensive washing with IPP buffer, high-salt wash buffer (500 mM NaCl, 10 mM Tris-HCl, pH 7.4, 0.1% NP-40), and low-salt wash buffer (50 mM NaCl, 10 mM Tris-HCl, pH 7.4, 0.1% NP-40), the RNA fragments were eluted from the beads by proteinase K (Roche, 3115836001) digestion at 55 °C for 1 h, phenol-chloroform extraction and ethanol precipitation. RNAs were recovered and subjected to library preparation using the KAPA RNA HyperPrep kit (KAPA Biosystems, KK8541). Sequencing were performed on an on Illumina HiSeq X-Ten platform with paired end 150 base pair (bp) read length. For MeRIP-qPCR analysis of the enrichment of m^{6}A peaks, equal amout of anti-IgG antibody(Beyotime, A7016) was also used for immunoprecipitation. Input RNA, IgG- and m^{6}A-IPed RNA was subjected to reverse transcription and subsequent qPCR. The relative enrichment of m^{6}A in each sample was calculated by normalizing to input. 18S rRNA was used as negative control. For MeRIP-qPCR analysis of the enrichment changes of m^{6}A peaks upon METTL3 deficiency, the relative enrichment of m^{6}A in siCTRL and siMETTL3 sample was calculated by normalizing to each input. The primers used were listed in **Supplementary Table S2**.

Sequencing data processing. The quality of all Illumina sequencing reads was firstly screened using FastQC (http://www.bioinformatics.babraham.ac.uk/projects/fastqc/) and then adaptor sequences were trimmed using the Cutadapt (version 1.18)[2]. Reads with length less than 35 nt or containing an ambiguous nucleotide were discarded by Trimmomatic (version 0.36)[3]. The remaining reads were aligned to the *Chlorocebus sabaeus* genome augmented with Ensembl (release 100)[4] genome annotation using

Histat2 (version 2.0.5)[5] with default parameters. And then the unmapped reads were aligned to the reference sequence of SARS-Cov-2 downloaded from NCBI (NC_045512.2)[6] using BWA (version 0.7.15)[7] with default parameters. For quantitative and comparative analysis, reads mapped to SARS-Cov-2 RNA (+) and SARS-Cov-2 RNA (-) were respectively extracted using Samtools (version 1.9)[8]. To minimize the rate of false positives, only uniquely mapped reads with -q ≥ 20 were kept for the subsequent analysis for each sample.

DAMS (DEW-based Analysis for MeRIP-Seq, DEW = Differential Expressed Window). The reference sequence (both *Chlorocebus sabaeus* and SARS-Cov-2) was first scanned using sliding windows of 25 nt. The coverage for each window was calculated for the MeRIP and input samples using Bedtools[9]. Using the R package edgeR[10], windows with $\log_2$ (fold change of MeRIP/input) > 2 and FDR < 0.05 were kept and then adjacent windows were merged. Only the merged windows with length more than 100 nt were finnally identified as m^{6}A peaks. Specially, compared to SARS-Cov-2, the depth of reads from *Chlorocebus sabaeu* is relatively low in this work so that we adjusted the cutoff as $\log_2$ (fold change of IP/input) > 2 and $p < 0.05$ for its m^{6}A peak calling.

MACS2. The host Vero cells m^{6}A-enriched peaks were also identified using MACS2 (version 2.1.4)[11] with the corresponding input sample as control. MACS2 was used with parameters '--nomodel, --keep-dup all and -g 2.8e9'. Peaks with FDR value < 0.05 and located in 5'UTR, CDS and 3'UTR of *Chlorocebus sabaeu* mRNA were kept for the following analysis. Only intersections between the peaks called by two replicates were used as final set of peak calls.

Differential m^{6}A peaks analysis. Peaks identified in SARS-Cov-2 using edgeR for control samples were used for subsequent differential analysis. The coverage for each peak was calculated for the MeRIP and input samples, which with or without siMETTL3 treatment, using Bedtools. Every peak was assigned the metric 1 (M1),

representing the enrichment fold change of MeRIP over input sample. The influence of RNA abundance and peak length in this method were normalized using RPKM. Differential m^{6}A peaks between control and siMETTL3 group were calculated using metric 2 (M2). Peaks were identified as METTL3-dependent in SARS-Cov-2 with $\Delta < -\log_2(1.5)$.

$$m^6A\ \text{enrichment}=\log_2\frac{\text{RPKM (MeRIP) of peak}}{\text{RPKM (Input) of peak}}\quad \text{(M1)}$$

$$\Delta = \text{Mean (m}^6\text{A enrichment, siMETTL3) - Mean (m}^6\text{A enrichment, control)}\quad \text{(M2)}$$

Motif analysis within m^{6}A peaks. Motif of m^{6}A peaks in *Chlorocebus sabaeu* were identified by HOMER (version 4.7)[12]. The sequence of peaks located in 5'UTR, CDS and 3'UTR of *Chlorocebus sabaeu* mRNAs were extracted as the target sequences and background sequences were obtained by randomly shuffling peaks upon total mRNAs on genome using BEDTools' shuffleBed (version v2.16.2)[9].

Permutation analysis for RRACH. To well identified whether m^{6}A peaks in SARS-Cov-2 were also RRACH dependent, the summit window of each peak was first defined by window with highest enrichment, which is calculated as $\log_2$ (fold change of MeRIP/input) using edgeR. For each peak, the center of summit window with up- and downstream 50 nt flanking sequences were scaned for RRACH. Finally, 11 m^{6}A peaks were validated in total 13 m^{6}A peaks along SARS-Cov-2.

Then the permutation analysis was performed to validate whether the enriched RRACH for m^{6}A peaks was randomly or not. For each time of permutation, the 13 peaks were first randomly shuffled along SARS-Cov-2 reference sequence and then the proportion of RRACH contained in random peaks were calculated. After 1,000 repeats, the observed propotions from the these data were contributed as empirical probability distribution, and only 74 simulated peak pools contained at least 11 RRACH peaks.

Counting and classifying reads for junction-spanning reads. The junction-spanning reads were categorized by the position of 5' and 3' site positions[13]. For each sample,

different junction-spanning types are estimated by the sum of junction-spanning reads. The junction-spanning type is considered as non-random if it contained more than $1/10^6$ of total junction-spanning reads.

Based on the location of its 5' and 3' site postions, the junction-spannings were then calssified into four groups, (a) TRS-L-dependent, canonical; (b) random 3' acceptor; (c) random 5' donor; (d) random inner junction (see **Fig. 1i**). For TRS-L-dependent, canonical, the 5' site position was aligned to SARS-Cov-2 genomic position from 71 to 78 and 3' was aligned at the upstream of each sgRNA CDS segment (S, ORF3a, E, M, ORF6, ORF7ab, ORF8, N). In our data , we found there were only one predominant junction-spanning for each sgRNA (see **Supplementary Fig. S3a and S3b**), and those predominant junction-spanning were then defined as the TRS-L-dependent, canonical. For random 3' acceptor, the 5' site positions were located around 71 to 78 nt along SARS-Cov-2 genome, but the 3' site positions were not at the predominant junction-spanning 3' site for each sgRNA. Similar to random 3' acceptor, junction-spanning of random 5' acceptor contained the predominant junction-spanning 3' site but the 5' site position is out of genomic position from 71 to 78 nt. Except that, the other junction-spannings were then classified as random inner junction. Especially, 3'UTR inner junctions were identified as the random inner junction whose 5' site position located in 3'UTR segment.

Expression of junction-spannings. The amounts of junction-spanning were first normalized by the ERCC for each sample (used in **Supplementary Fig S2c, S3a and S3b**). To well compared the propotions of different junction-spannings in each samples, the expression level of junction-spannings were valued as RPM (reads per million) by normalized with the sum of total junction reads (used in **Fig 1h and 1k and Supplementary Fig. S3d**).

Statistical analysis. All statistical analyses of qPCR assay were performed two or three independent experimental replicates. Student's two-sided unpaired *t*-test was used for statistical comparisons and data were shown as mean ± S.E.M.

References

1. Cross, S. T. *et al*. RNA regulatory processes in RNA virus biology. *Wiley Interdiscip Rev RNA* **10**, e1536, doi:10.1002/wrna.1536 (2019).
2. Martin, M. Cutadapt removes adapter sequences from high-throughput sequencing reads. *EMBnet J* **17,** 10–12 (2011).
3. Bolger, A. M., Lohse, M. & Usadel, B. Trimmomatic: a flexible trimmer for Illumina sequence data. *Bioinformatics* **30,** 2114–2120 (2014).
4. Kim, D., Langmead, B. & Salzberg, S. L. HISAT: a fast spliced aligner with low memory requirements. *Nat Methods* **12**, 357-360 (2015).
5. Wu, F. *et al*. A new coronavirus associated with human respiratory disease in China. *Nature* **579**, 265-269 (2020).
6. Li, H. & Durbin, R. Fast and accurate short read alignment with Burrows-Wheeler transform. *Bioinformatics* **25**, 1754-1760 (2009).
7. Li, H. *et al*. The Sequence Alignment/Map format and SAMtools. *Bioinformatics* **25**, 2078-2079 (2009).
8. Quinlan, A. R. & Hall, I. M. BEDTools: a flexible suite of utilities for comparing genomic features. *Bioinformatics* **26**, 841-842 (2010).
9. Robinson, M. D., McCarthy, D. J. & Smyth, G. K. edgeR: a Bioconductor package for differential expression analysis of digital gene expression data. *Bioinformatics* **26**, 139-140 (2010).
10. Zhang, Y. *et al*. Model-based analysis of ChIP-Seq (MACS). *Genome Biol* **9**, R137 (2008).
11. Heinz, S. *et al*. Simple combinations of lineage-determining transcription factors prime cis-regulatory elements required for macrophage and B cell identities. *Mol Cell* **38**, 576-589 (2010).
12. Kim, D. *et al*. The Architecture of SARS-CoV-2 Transcriptome. *Cell* **181**, 914-921 e910 (2020).
